# Probing the Nanodomain Origin and Phase Transition Mechanisms in (Un)Poled PMN-PT Single Crystals and Textured Ceramics

**DOI:** 10.3390/ma3125007

**Published:** 2010-11-25

**Authors:** Aneta Slodczyk, Philippe Colomban

**Affiliations:** Laboratoire de Dynamique, Interactions et Réactivité (LADIR), UMR 7075, CNRS, UPMC, 2 rue H. Dunant 94320 Thiais, France; E-Mail: aneta.slodczyk@glvt-cnrs.fr

**Keywords:** PMN-PT, ferroelectric relaxor, piezoelectric, Raman scattering, solid solutions

## Abstract

Outstanding electrical properties of solids are often due to the composition heterogeneity and/or the competition between two or more sublattices. This is true for superionic and superprotonic conductors and supraconductors, as well as for many ferroelectric materials. As in PLZT ferroelectric materials, the exceptional ferro- and piezoelectric properties of the PMN-PT ((1−x)PbMg_1*/*3_Nb_2*/*3_O_3_−xPbTiO_3_) solid solutions arise from the coexistence of different symmetries with long and short scales in the morphotropic phase boundary (MPB) region. This complex physical behavior requires the use of experimental techniques able to probe the local structure at the nanoregion scale. Since both Raman signature and thermal expansion behavior depend on the chemical bond anharmonicity, these techniques are very efficient to detect and then to analyze the subtitle structural modifications with an efficiency comparable to neutron scattering. Using the example of poled (field cooling or room temperature) and unpoled PMN-PT single crystal and textured ceramic, we show how the competition between the different sublattices with competing degrees of freedom, namely the Pb-Pb dominated by the Coulombian interactions and those built of covalent bonded entities (NbO_6_ and TiO_6_), determine the short range arrangement and the outstanding ferro- and piezoelectric properties.

## 1. Introduction

### 1.1. Heterogeneity and electrical properties: solid solution a wrong concept

In the solid state, most of the outstanding electrical/electrochemical/magnetic properties result from a competition between different potentials of the chemical bonding and structure. In many cases, some atoms (alkali and earth alkati cations, Pb^2+^, Ag^+^, *etc*.) have a strong ionic character and develop long range Coulombian interactions. Some other atoms develop covalent bonding (e.g., transition metals, *etc*.) and form strong “molecular” bricks. Metallic bonds can also be formed between atoms such as Ag, Cu, Tl, and ionized clusters intermediately between ions and complex nanoclusters may be observed [1,2,3]. Thus, in a crystal, as a function of the crystalline axis, different types of bonds can simultaneously be present. This discovery was first made for KNiF_4_ by J.B. Goodenough and is the origin of supraconductivity [4,5,6,7].

Another source of heterogeneity is the multielemental composition. The mixing of different (here we will limit our attention to oxides) raw materials, according to the phase diagram may lead to precipitation of different phases as a function of the raw materials used, the thermodynamics (the pathway to more stable phase must be blocked at a certain temperature as a function of starting reagents), the heating/cooling rates, and annealing time. In some cases, the homogeneity looks perfect to the eye and the material was considered—in the past—as homogeneous, *i.e.,* for some glasses and solid solutions [8,9]. Eutectics presents an intermediate situation [10,11], the phase coexistence being expected to be repeated from visible to short scale. Actually, if we consider a solid solution, we can calculate the mean distance between substituted atoms and obviously, at this scale, the material is no more a solid solution but a multiphased material. Furthermore, at the limit between these two phases, the atoms undergo a chemical field and bonding that are different from those present in the phase core. The situation is, thus, very similar to the difference observed between the core/bulk and the surface (actually near the surface) of nanoparticles. In conclusion, a compound previously described as homogeneous is actually a three-phase mixture and should be analyzed with appropriate techniques.

For a long time, the extraction of information about short range structure has required the study of X-ray and neutron diffuse scattering, which needs (big) single crystals and particular conditions and/or composition to get a good contrast. The best results were obtained for 1-dimensional (1-D) or 2-D structures [3]. Consequently, the number of compounds that satisfy experimental requirements are scarce and invstigation of the problem of the competition between short range ordering and long range mean structure remains limited to a small community of scientists. The recent impetus for the understanding and optimization of materials at the nanoscale enlarges the scope and neutron facilities allow study of diffuse scattering, especially in perovskites [12,13]. It should be noted that among the interactions of light with matter, Raman scattering is particularly well suited to multiscale analysis of ill-organized heterogeneous solid as the corrosion films [14,15,16,17]. The Raman probe offers a “bottom-up” approach to nanomaterials and amorphous compounds, which works best in the case of imperfect crystals built of strong covalent bonds [14].

It is clear that the concept of solid solution is rarely valid for the understanding of electrical properties and associated solid state physics and chemistry. The intrinsic short range heterogeneity of solid solution makes the boundary between two symmetries not necessarily straightforward, and as for using the fractal concept, the length of an irregular boundary depends on the scale length. The limit between two symmetries will also depends on the observation scale.

### 1.2. The perovskite structure

**Figure 1:**
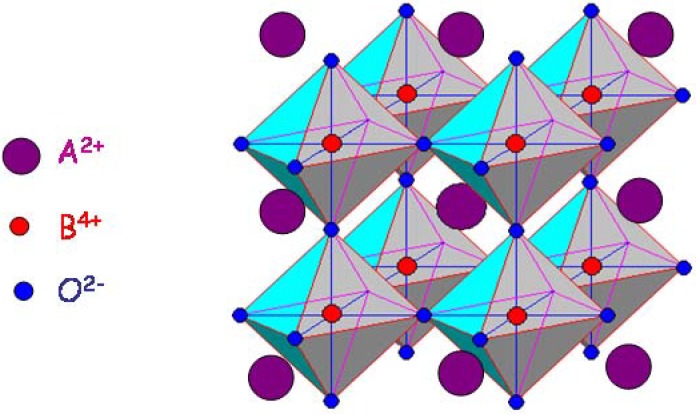
Perovskite structure made of two sublattices: 3-dimensional network of corner linked BO_6_ octahedra and cubic A^2+^ network.

Many compounds belonging to the Perovskite-like structure family present outstanding electrical properties [18,19,20]. CaTiO_3_ is the natural mineral presenting the perovskite structure; however the reference compound is BaTiO_3,_ the synthetic compound at the base of the dielectric industry [19,20,21,22,23]. The general formula of a perovskite is A^2+^B^4+^O^2−^_3_. The ideal perovskite structure presented in Figure 1 possesses the cubic symmetry with the Pm3m space group and is made of two sub-lattices: (i) a covalent-bonded network of BO_6_ octahedra sharing their oxygen atoms with the B-ion at the center, and (ii) the lattice of A cations, larger than the B cations, placed in the space between octahedra for which long distance Coulombian interactions are important. The competition between the degree of freedom of each sublattice leads to different structural variants such as atom displacements and/or octahedral tiltings [18,24,25,26]. Consequently, more or less important distortions from the ideal cubic structure are observed. Such distortions can be limited to the local symmetry: nanoregions, nanodomains (for example BaZrO_3_ [26,27,28]) or can give rise to the long range order structural phase transition sequence (for example BaCeO_3_ [29,30]). Displacive phase transitions in titanates, cerates and associated systems have been extensively studied by Raman and neutron scattering for a long time [21,22,23,29,30,31,32,33,34,35].

In the case of many perovskites, the A and B sites are substituted by different ionic species with different valences and ionic radii. Consequently, the so–called “perovskite solid solution”, with a general formula of A’_1−x_A”_x_B’_1−x_B”_x_O_3_, appears. If the added atom does not have the same valence, structural defects such as vacancies are formed to satisfy the charge equilibrium. Such a phenomenon is widely observed in the case of perovskite showing the ion conduction where the partial substitution of the B site by the lower valance ion leads to the appearance of the oxygen vacancies [26,36 and refs therein]. If the substitution levels is small, the interdistance between vacancy and substituted cation increases to ~10 nm scale, *i.e*., the scale required to permit the condensation of diffuse Bragg peaks into (small) superstructure peaks [24,25]. For a high level of substitution, different variants such as double perovskites, Ruddleson-Popper, Aurivillius, Dion-Jacobson phases, *etc*. [37,38,39,40,41,42,43,44], in which segregation of vacancies can lead to an empty plane, may be formed and extra/superstructure diffraction peaks are clearly observed. This gives rise to a great variety of properties: optical, magnetic, electric, mechanical, conducting, *etc*., causing these compounds to be the subject of intensive studies for decades. It should be stressed, however, as noted by Roy and co-workers [19], that these properties are not directly dependent on the structure. Perovskite proves the (common) error of the structure-property *cliché* widely presented in the literature.

### 1.3. Relaxor ferroelectrics—Perovskite “solid solutions”

Among the most studied perovskite *solid solutions*, one can easily place the different relaxor ferroelectric solid solutions [45,46,47,48,49,50,51,52,53,54,55,56,57,58,59,60,61,62,63,64,65,66,67,68,69,70,71,72,73,74] such as PbZr_1−x_Ti_x_O_3_ (PZT) [48,53,54], (Pb,La)Zr_1−x_Ti_x_O_3_ (PLZT) [55,56], (1−x)Pb(Mg_1/3_Nb_2/3_)O_3_–xPbTiO_3_ (PMN-PT) [57,58,59,60,61,62,63,64,65,66,67] and (1−x)Pb(Zn_1/3_Nb_2/3_)O_3_−xPbTiO_3_ (PZN-PT) [67,68,69,70,71,72,73,74]. Note that we will use *solid solutions* formula in this article as is general in the literature; however, one should use this with caution, since as we will show, the studied materials are not really solid solutions at the scale determining most of their properties! When properly orientated, relaxor ferrolectrics exhibit extremely large values of the dielectric constant, giant piezoeffect, ultrahigh strain and electrostriction [45,46,47,48,50,51,72,75,76,77]. These properties make them very interesting candidates for medicine, telecommunication, high-tech and military devices. However, prior to such industrial application, one has to understand/clarify their complex physical and chemical behavior.

The relaxor ferroelectric solid solutions exhibiting the giant piezoeffect are the Pb-based perovskites, which include at the B-site different quantity of ionic species with different valences and ionic radii: two (Zr and Ti) for PZT and three (Ti, Mg and Nb) in the case of PMN-PT. The charge and ionic size differences are very important, because they directly determine the degree of order in the B-ion sublattice and then the coherence length of order [78,79,80,81]. Material with a long coherence length of order behaves as a normal ferroelectric, *i.e*., PbTiO_3_ [45,46,47,51]. On the contrary, the relaxor behavior is characteristic for solid solutions in which the (random) distribution of the B-ions or the existence of nanoregions with short coherence length of order is observed [45,47,49]. Moreover, the random distribution of the B-ions is the source of the random electric fields and random mechanical stresses. This prevents the development of the long range ferroelectric order. Therefore, in relaxors the polar order is limited to the nanoscale and as an effect, so–called polar nanoregions (PNRs) are postulated [52,82,83]. Both the chemically ordered clusters and the polar nanoregions can exhibit distinct local symmetry. The nanoregions embedded in the disordered cubic matrix seem to play a key role in the physics of ferroelectric relaxors.

**Figure 2:**
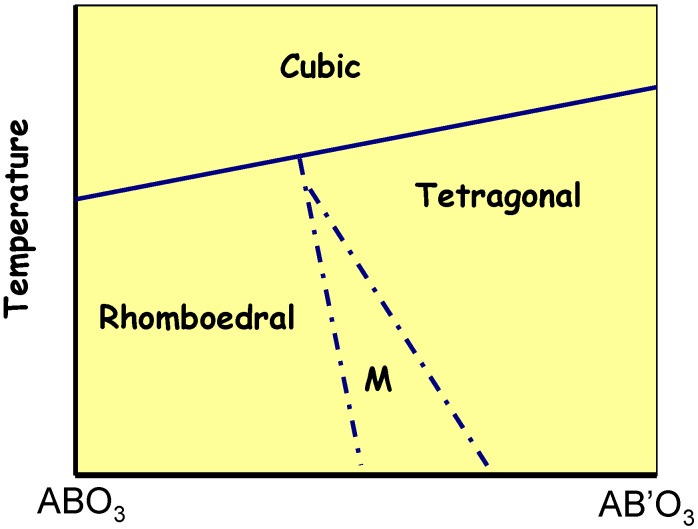
Phase diagram of a “solid solution” with a morphotropic boundary region (M).

Consequently, the phase diagrams of the Pb(B’,B”)O_3_-PbTiO_3_ systems are very complex [48,50,57,58,59,60,61,62,63,64,65,66,67,68,69,72]. As it is sketched in Figure 2, at a high temperature, above the Curie temperature, cubic, para-electric symmetry is observed by X-ray diffraction. Below a temperature close to 600 °C (PZT, PLZT), 400–500 °C (PZN-PT or PMN-PT), [48,50,55,56,57,58,59,60,61,62,63,64,65,66,67,68,69,72,84,85,86,87,88,89], the so–called rhombohedral–relaxor side specific for materials with a small PT/high PB amount and the tetragonal–ferroelectric one characteristic for solid solutions with a high content of PT/low content of PB can be distinguished. Obviously, for one specific composition, the structure may have three symmetries: this requires specific ratios between unit-cell parameters. Both these sides are separated by the so-called morphotropic phase boundary (MPB). It is not clear if the exact boundary between the symmetry range depends on the scaling of the analyzing method used: X-ray/neutron/electron diffraction or Raman/neutron scattering.

The concept of the morphotropic phase boundary was introduced for the first time by Jaffe *et al*. [48] in the case of the PbZr_1−x_Ti_x_O_3_ system and was described as an almost vertical boundary, nearly temperature independent, between the rhombohedral and tetragonal phases. Further, some authors showed the MPB to be the region in which the low symmetry phases, *i.e*., monoclinic or orthorhombic, are present [50,60,63,66,67]. Actually, the MPB should be considered as the region in which the phases with different length and type of order can coexist. Moreover, such coexistence of the energetically close phases seems to play the key role in appearance of the giant piezoelectricity in this system. Namely, the monoclinic structure has the unique property that it allows the polar direction to rotate within the basal plane; this freedom stands in sharps contrast to the uniaxial constraint imposed on the polar direction in both R and T symmetries [77,84,85,86,87].

### 1.4. Anharmonicity, phase transition and vibrational signature

The above description clearly shows that the physical/chemical behavior of the relaxor ferroelectric solid solutions is very complex. The richness of phase transitions, coexistence of phases with different coherence length, coexistence/competition of relaxor and ferroelectric behavior lead to many discrepancies and contradictions. Despite intensive studies, the physical and electrochemical properties directly connected with the relaxor ferroelectric solid solutions are still not fully understood. Since the physics of these systems takes place mainly on the nanoscale, the Raman spectroscopy that probes the local structure at the very short range is one of the most useful techniques to study these compounds [14,15,16,17,54,60,61,62,63,70,73,74]. Moreover, this technique studies directly the chemical bond anharmonicity, which allows—especially if coupled with the thermal expansion measurements as we will show below—detection of any very subtle structural modifications that are overlooked by many studies.

The potential anharmonicity is very important as far as the phase transition is concerned. As a rough description, a strong bond anharmonicity promotes the easy displacement of one atom from its equilibrium position in its site toward another position, and hence is at the origin of (super)conductivity (efficient ion jump) and of displacive phase transition [33,36]. The highest anharmonicity is observed with a proton, because of the huge elasticity of the X-H…Z bond [36 and refs therein]. Anharmonicity and dielectric properties are correlated because the anharmonic instant displacement of ions creates instant dipole and complex impedance measurements in the MHz-GHz range, allowing characterization of the different motions.

Thermal expansion *vs.* temperature is another direct measurement of the bond anharmonicity and measurements along the different directions of a single crystal (or highly textured ceramic) give information about the (3D) anisotropy. Plots of the IR or Raman wavenumber *vs.* temperature, *vs*. pressure, or *vs*. stress also give information about the anharmonicity—wavenumber and bond distance are independent of the temperature and pressure in harmonic material [88,89].

From the vibrational point of view, the Raman signature of a covalent-bonded structure can be described as that of the covalent entities constituting the structure (BO_6_ octahedron). The other ions are highly ionic and contribute with the translation oscillation modes (T’) (low energy, below ~250 cm^−1^), which couple themselves with other vibrational modes of the neighboring entities: translations (T’) and rotations/librations (R’) of the iono-ocovalent BO_6_ entity leading to the wavenumber shifts. A low wavenumber region (100–250 cm^−1^) chiefly involves a cationic network and the lattice modes, while a 250 to 800 cm^−1^ spectral range reveals the bending and stretching modes of the covalent-bonded octahedron [26,38,60,61,62,90].

In this paper, we will show, using specific cases, that Raman spectroscopy is a good tool to document the structure, symmetry and short range order at the very nanoscale. As the Raman probe is for interatomic bonds themselves, the technique offers a “bottom-up” approach to study nanosized materials and amorphous compounds, which works best in the case of lattices with strong covalent bonds and high Z number atoms such as those present in perovskite compounds. The Raman results will be compared with those of thermal expansion measurements. We will use, as an example, two PMN-PT compositions from the MPB region: FC (field-cooled) poled and unpoled single crystal (SC) PMN-29%PT as well as RT (room-temperature) poled and unpoled highly textured ceramic (HTC) [60,61,62,91,92,93] PMN-34.5%PT. The polarization kind can change/modify the complex sequence of the structural phase transition of both systems, and the coupling of Raman scattering and thermal expansion analysis is power tool to detect and then to understand these modifications.

## 2. Results and Discussion

### 2.1. Phase transitions by dielectric and thermal expansion measurements

**Figure 3:**
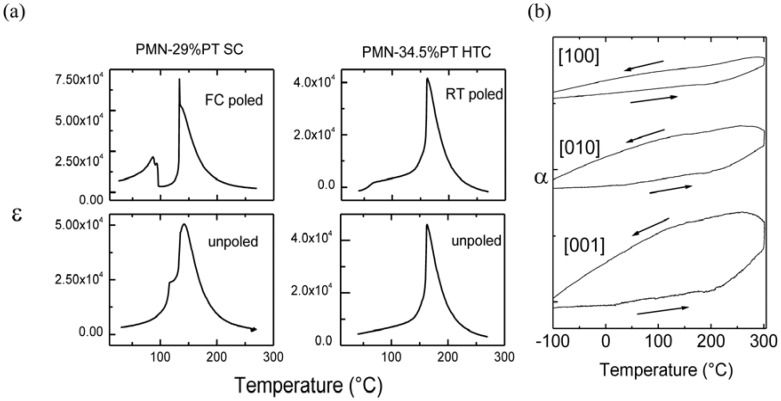
(**a**) Dielectric constant *vs*. temperature for FC poled and unpoled PMN-29%PT single crystal as well as RT poled and unpoled PMN-34.5PT highly textured ceramic; (**b**) Anisotropy of thermal expansion curves observed for highly textured ceramic [60,61,62].

Figure 3a summarizes the temperature dependences of dielectric permittivity for poled and unpoled: PMN-29%PT single crystal and PMN-34.5%PT textured ceramic. As it can be seen, the dielectric permittivity curves are different for poled and unpoled materials, which clearly proves the important influence of the polarization type on the structure and physical behavior. FC poled PMN-29%PT single crystals exhibit two main maxima of dielectric permittivity: a strong one observed at 135 °C and a less intense, rather doubled near 90 °C. Both maxima exhibit the ferroelectric character. It should be stressed that the main peak detected at 135 °C seems to be rather a superposition of a very intense and very narrow one, and the other is less intense and broader, suggesting the structural heterogeneity. In the case of unpoled PMN-29%PT, two dielectric permittivity maxima are also detected. However, the temperature of the maximum values are different, namely 120 °C and 140 °C, and the curves have a rather relaxor character [45,46,47]. This suggests that the ferroelectric state can be achieved after the FC polarization only. Two maxima of dielectric permittivity are also recorded for the RT poled PMN-34.5%PT textured ceramic: a strong and sharp one at 160 °C, rather similar to that observed for the FC poled PMN-29%PT, and the second one is more smoothed and broad, near 70 °C. The unpoled ceramic exhibits only one main maximum of dielectric permittivity, near 160 °C. As it can be seen, this maximum is very similar to that characteristic for a poled sample. This shows that the temperature of the main maximum of dielectric permittivity seem to be almost independent of the poling conditions proving the true ferroelectric state and requires very accurate techniques like thermal expansion to detect the differences. Note that in the case of relaxors, the kind of polarization significantly changes the temperature and character of phase transitions, whereas for the ferroelectrics the role of polarization seems to be less important [62].

As can be clearly seen in Figure 3a, the maximal values of dielectric permittivity are very high, both for single crystals and textured ceramic, which is characteristic for PMN-PT solid solution from the MPB region. It is, however, noteworthy that the values recorded for textured ceramics are almost comparable with those of single crystals. Moreover, the thermal expansion measurements shown in Figure 3b reveal that the anisotropic character of the textured ceramic, namely the thermal expansion curves recorded along the x, y, z directions of cut ceramic, close to the [100], [010] and [001] crystallographic directions, is different [62]. This clearly shows the application potential of the textured ceramics and makes them a low cost alternative of the single crystal.

**Figure 4:**
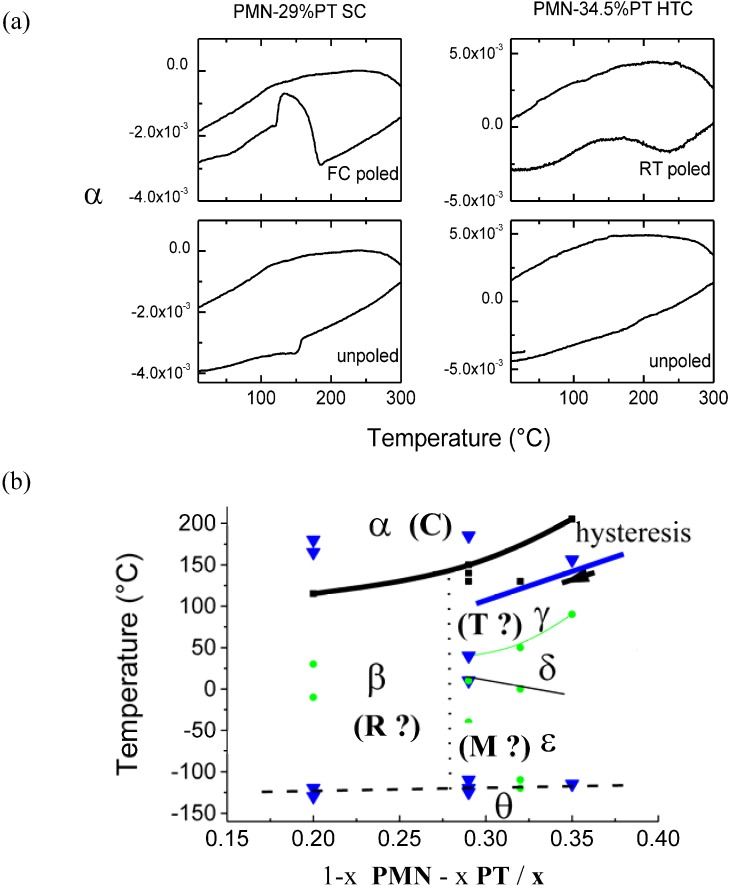
(**a**) Thermal expansion curves for poled and unpoled PMN-29%PT single crystal and PMN-34.5%PT textured ceramic; (**b**) PMN-PT phase diagram determined due to the thermal expansion results [61]. The symbols α–θ mark the phases of different symmetry, which complete these ones determined by X-ray diffraction (cubic C, rhombohedral R, tetragonal T and monoclinic M). Note that the presence of the temperature hysteresis allows classification of some of these transitions as first order ones. The arrows mark the transition line on a cooling cycle.

In the case of materials from the MPB region, the maxima of dielectric permittivity reveal the presence of phase transitions. In order to better understand these modifications, the thermal expansion data were carefully analyzed [61,62]. Figure 4a presents the thermal expansion curves characteristic for poled and unpoled single crystal and textured ceramic.

As can be seen in Figure 4a, the thermal expansion curves show, in general, the anomalies near similar values of temperatures determined by the dielectric results (the small temperature shifts are attributed to the different heating rates). In the case of FC poled single crystal, two main transitions are detected near 90 °C and 135 °C. The observation of a sharp expansion jump and then a smooth decrease is consistent with the dielectric feature observed near 135 °C (see Figure 3a). Note that dilatometry results point to the presence of two additional weak phase transitions near 40 and 180 °C and at a lower temperature as we presented in our previous work [61]. Similar behavior is observed for RT poled highly textured ceramic: two transitions near 70 and 160 °C, already detected by dielectric measurements, are well seen, whereas the thermal expansion curve reveals the presence of another weak transformation in the vicinity of 200 °C. The dilatometry data also confirm the phase transitions for both unpoled samples, as it can be seen that these transitions do not significantly change the unit cell volume and have smooth character. Due to the thermal expansion results performed in a wide temperature range [61], a more complex phase diagram has been proposed (Figure 4b). As it can be seen, these data point to high complexity of the phase transition sequence in the MPB range (see Table 1).

Unambiguous interpretation of these observed phase transitions is rather difficult. The richness of phase transitions, coexistence of phases with different length of scale and competition between relaxor and ferroelectric behavior are typical for solid solutions from the MPB region. Consequently, many phase diagrams have been proposed [57,58,59,60,61,62,63,64,65,66,67] as a function of selected samples and/or used techniques. The structural modification connected with the main, strongest maximum of dielectric permittivity seems to be the easiest to interpret. The phase diagrams show very good agreement. Therefore, the main phase transition observed at 135 °C and at 140 °C for the FC poled and unpoled single crystals, respectively, can be attributed (according to the diffraction data [57,58,59,63]) to the transition from the rhombohedral symmetry to the cubic paraelectric one. Similar, the main transitions detected near 160 °C in the case of RT poled/unpoled texture ceramic might be assigned to the transition from tetragonal to cubic symmetry [57,58,59,63]. Note that no or very little hysteresis is observed by thermal expansion for compositions with x < 0.3. The origin of the additional phase transition detected at lower temperatures is more complicated. FC poled single crystal shows two structural modifications at 90 °C and then 40 °C, whereas the unpoled one undergoes one transition at 118 °C. According to the widely accepted Noheda’s phase diagram [59], PMN-29%PT possesses rhombohedral symmetry and does not exhibit any low temperature phase transition. However, some authors clearly show the presence of such additional phase transition(s) in the solid solutions with similar compositions: 27%PT, 28%PT and 29%PT [63,66,67]. These authors interpreted this as structural phase transitions from monoclinic (Cm or Pm space group) to rhombohedral symmetry. Moreover, even the presence of structural phase transitions between two different monoclinic symmetry and/or the coexistence of the phases (two monoclinic or rhombohedral-monoclinic) were suggested [66,67]. RT poled PMN-34.5%PT shows clearly the structural phase transitions at 70 °C. According to the Noheda phase diagram [59], such transition can be explained as the transformation from low temperature monoclinic symmetry to tetragonal. Recently, X-ray diffraction study has shown that the single crystal with 35%PT [63] rather exhibits coexistence of monoclinic and tetragonal phases at low temperatures.

Especially controversial is, however, the presence of phase transitions detected by thermal expansion measurements above the maximum of dielectric permittivity for both poled compounds [61]. According to the literature, at temperatures above the maximum of dielectric permittivity, the symmetry observed by X-ray diffraction is cubic; as a consequence, no further phase transition is expected [57,58,59]. In our opinion, taking into account the extremely high sensitivity of thermal expansion technique to detect a structural modification, these “very high” temperature phase transitions can be attributed to the transition from cubic symmetry with polar nanoregions (PNR) to the “true” cubic symmetry. As it is known, the polar nanoregions appear at the so-called Burns temperature [52], and X-ray diffraction, as well as the dielectric measurements, do not detect their presence. The symmetry is considered to be cubic (Pm3m); however, according to the local scale measurements, the presence of the polar nanoregions embedded in the nonpolar cubic matrix was postulated [52,63,82,83] (see Figure 7).

The thermal expansion and dielectric results clearly show that the poling conditions modify the sequence of phase transitions for both the PMN-29%PT single crystal and PMN-34.5%PT textured ceramic. The comparison of the observed phase transition in the 20–250 °C temperature range for poled and unpoled samples is presented in Table 1.

**Table 1:** Phase transition in the 20–250 °C temperature range detected for poled and unpoled PMN-29%PT single crystal and PMN-34.5%PT highly textured ceramic.

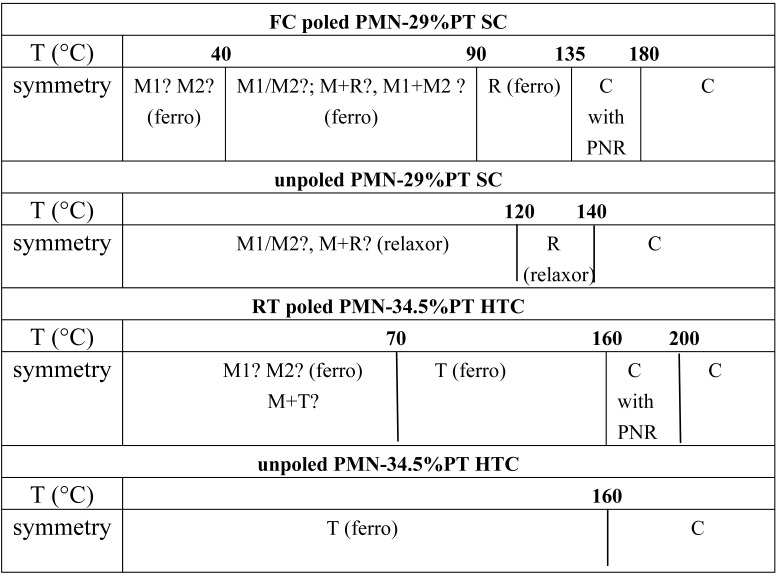

### 2.2. Raman studies *vs.* temperature

**Figure 5:**
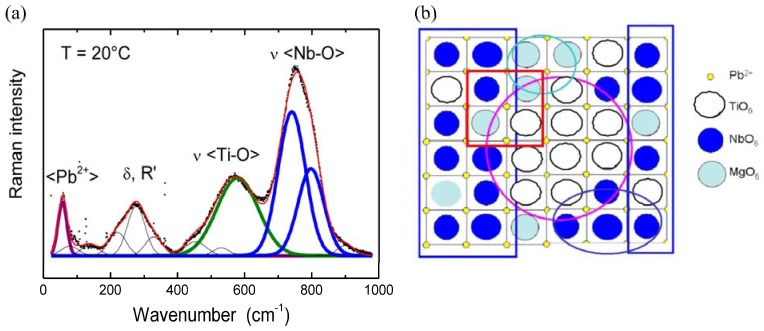
(**a**) Example of Raman spectrum decomposition and mode assignment according to the used sublattice model: ν stretching BO_6_ modes, δ bending modes, R’ librations and <Pb^2+^> translations of Pb^2+^; (**b**) Sketch of the very local distribution of the different BO_6_ octahedra: at least four domain types are present: Ti-based, Mg-based, Nb-based and region(s) at the boundary between “pure” ones.

The Raman spectra of both poled and unpoled PMN-29%PT single crystal and PMN-34.5%PT highly textured ceramic look at a first view very similar to those usually observed for relaxor ferroelectric samples [54,60,61,62,63,70,73,74,94,95,96]. They consist of a number of broad bands observed over the whole measured temperature range. In the “as-measured” form, these Raman spectra do not change much with the temperature increase. In order to determine the quantitative changes of the Raman mode parameters (wavenumber, width and intensity) *versus* temperature, the spectra were first corrected by the Bose-Einstein population factor and then carefully decomposed. [60,61,62]. Figure 5a presents, as an example, the decomposed and corrected room temperature Raman spectra characteristic for poled PMN-34.5%PT ceramic.

It is noteworthy that the Raman spectra are presented even within the paraelectric cubic phase with the Pm3m space group. For such cubic symmetry, the presence of first order Raman signature is not allowed [28,63,90]. One should note, however, that the origin of the observed Raman spectra, even at low temperature, remains unclear. As we have already mentioned in Section 1, the PMN-PT solid solution is characterized by the coexistence of polar nanoregions and chemically ordered clusters (Figure 5b), which is a source of short to long range intrinsic disorder. Note that the high structural disorder is directly responsible for very broad bands [14,28]. The eventual presence of the polar nanoregions (PNR) with the distinct local symmetry different than this cubic one, due to the ions being displaced from their high mean symmetry positions, give rise to the local electric dipoles [60,61,62,63,70,73,74,94,95,96] and the local distortions from cubic symmetry allow the Raman activity. The presence of chemically ordered clusters additionally complicates understanding of the Raman spectrum. There are three different ions at the B site: Nb, Mg and Ti (see Figure 5b). In general, in the case of a compound made of domain (with size smaller that the laser spot, °0.5 µm) with a different composition, it is impossible to determine if the recorded Raman spectrum comes rather from the pure or mixed domain. As shown in Figure 5b, in the case of the investigated PMN-PT, at least four domain types are present: Ti-based, Mg-based, Nb-based and region(s) at the boundary between “pure” ones, and each of them can give a Raman spectrum; the higher its distortion from cubic symmetry, the higher its Raman intensity. Moreover, since the Raman cross section depends mainly on the bond polarizability, *i.e*., the number of electron involved in the bond, it is highly possible that the Raman spectra will not reflect the signature of all regions [14,17]. In the case of PMN-PT, the following values of the Raman mode intensities are expected: Nb-O > Ti-O >> Mg-O. As a consequence, the contribution of Mg-rich regions is too weak to be observed, as far as the bending and stretching modes are considered. Consequently, the unambiguous Raman mode assignment is rather delicate. According to the previous attempts of mode assignment [60,61,62,63,92,93,94], as well as by the comparison with the Raman signatures of other perovskites [88], the low frequency region can be attributed to the vibrations of the Pb^2+^ sublattice; the modes from the intermediate frequency region can be classified as mixed B-O-B bending modes, while those from the high frequency region as BO_6_ stretching vibrations (Figure 5a).

#### 2.2.1. Temperature dependences of Raman mode parameters

**Figure 6:**
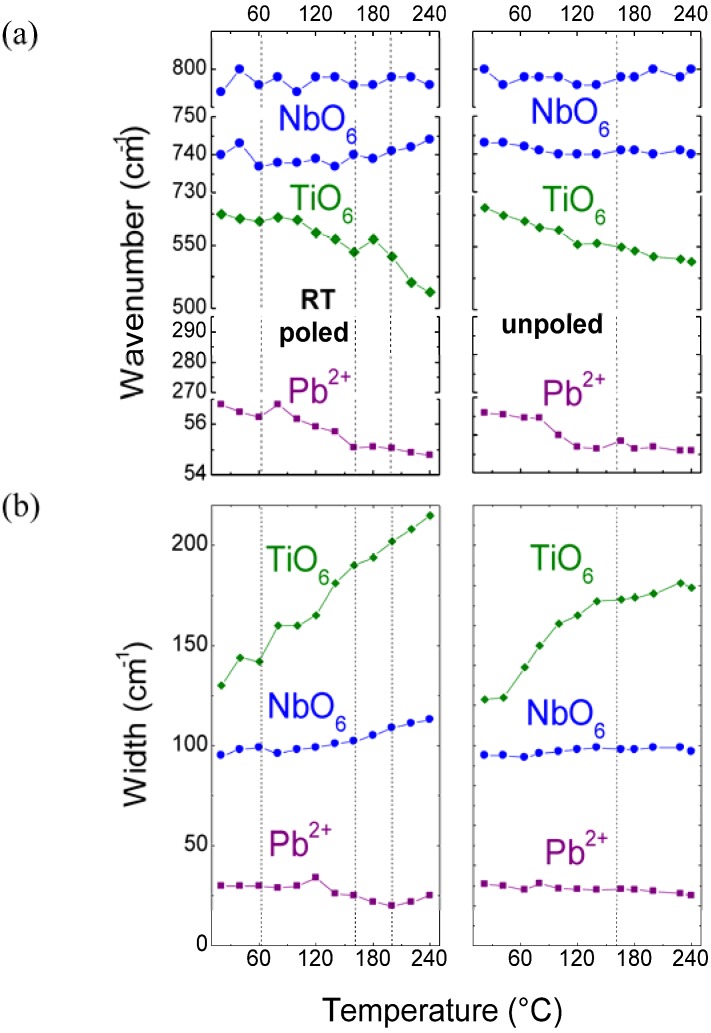
Temperature dependences of Raman mode wavenumbers (**a**) and widths (**b**) for RT poled and unpoled highly textured ceramic. The dashed lines represent the phase transition temperature determined from the dielectric and thermal expansion measurements. Note that since the heating rates are very different for Raman scattering and other techniques a shift of a few degrees is likely [62].

The temperature evolutions of Raman mode parameters are very similar for PMN-29%PT and PMN-34.5%PT. Therefore, the comparison between the poled and unpoled state will be restricted to the texture ceramic only. The temperature dependences of the wavenumbers for some selected Raman modes characteristic for poled and unpoled PMN-34.5%PT are presented in Figure 6a. Note that the modes presented in Figure 6 were selected in order to follow the behavior of characteristic sublattices/regions (Figure 5a).

As it can be seen in Figure 6a the wavenumber of the mode present near 50 cm^−1^ assigned to the Pb ion sublattice vibrations (Figure 5b) decreases with increasing temperature, achieving the minimal values near the phase transition temperatures. This provides evidence that the presence of the “step transitions” of the Pb^2+^ ion sublattice according to the unit cell parameters changes. This straightforward phenomenon is observed for RT poled ceramic. If we take into account that generally, in the “classical case”, a wavenumber value decreases slightly (a few cm^−1^) with increasing temperature due to the thermal expansion effects, the behavior of this mode is “rather” classical. In contrary, the temperature evolutions of modes located near 570 cm^−1^ (TiO_6_ stretching vibrations) and those *ca.* 800 cm^−1^ (NbO_6_ stretching vibrations) are not standard. The wavenumber characteristics of the 570 cm^−1^ TiO_6_ octahedron mode exhibits strong temperature sensitivity. Namely, first, the wavenumber decreases strongly by about 40 cm^−1^ and then anomalously increases above the maximum of dielectric permittivity (~170 °C). Such non-standard behavior is particularly well seen in the case of the poled sample. Note that this maximum at 170 °C corresponds well to the additional phase transition recorded during the thermal expansion measurements. The wavenumbers of modes located near 800 cm^−1^ do not vary much with increasing temperature. The temperature dependences of the wavenumbers thus show that the NbO_6_ octahedra are stable during temperature increases, while those of TiO_6_ soften continuously. This can be related to the larger size of NbO_6_ moieties that play the role of stable pillars for the structure.

The temperature dependences of the full width at half maxima (FWHMs) for poled and unpoled PMN-34.5%PT solid solution are presented in Figure 6b. As we have already mentioned, typically for relaxor ferroelectric solid solutions the Raman modes are rather broad, especially those characteristic of internal BO_6_ modes observed in the middle and high frequency range, *i.e*., ~60 cm^−1^ at room temperature. It is noteworthy that the widths of external, low wavenumber modes related to the Pb sublattice vibrations measured are rather standard, *i.e*., ~20 cm^−1^. This difference shows directly that the structural (orientational) disorder is connected to the octahedral sublattice because of the presence of different elements (see Figure 6) with different iono-covalent character in the B-site, *i.e*., covalent Ti and Nb atoms, and ionic Mg.

The FWHMs of modes located near 50 cm^−1^ (Pb^2+^ translational vibrations) do not change very much with temperature. Surprisingly, the highest values of the FWHM are not observed at the highest temperature but close to the temperature characteristic for structural phase transitions. This is especially well seen for the RT poled sample. We can expect that this is associated to some displacive disorder. However, similar FWHM behavior was already detected for PMN-PT solid solutions [63,94,96] and attributed to the Pb ion off-centered displacements: disordered or correlated for the short range scale (nanodomains). This points to the important role of the Pb ion sublattice in the physical behavior of the PMN-PT system.

The FWHMs of the modes located near 800 cm^−1^ increase very slightly with temperature increase. Such behavior is in very good agreement with the temperature dependences of the wavenumber and confirms well the high stability of the NbO_6_ octahedra sublattice. On the contrary, the widths of modes located near 570 cm^−1^ attributed to the TiO_6_ stretching vibrations increase enormously, particularly for poled ceramic. This reveals that the high level of structural disorder strongly affects the behavior of TiO_6_ octahedra. Note that the abrupt increase of the FWHM is detected in the vicinity of the low temperature phase transition, *i.e*., 70 °C, showing well the high sensibility of the Raman mode parameter in detecting structural modifications. Our results clearly show the significant differences between the temperature behavior of the FWHMs of the Pb^2+^ translational modes and those attributed to the BO_6_ stretching vibrations—behavior characteristic for perovskite materials—in perfect agreement with our description in terms of two independent sublattices [14,37,38].

**Figure 7:**
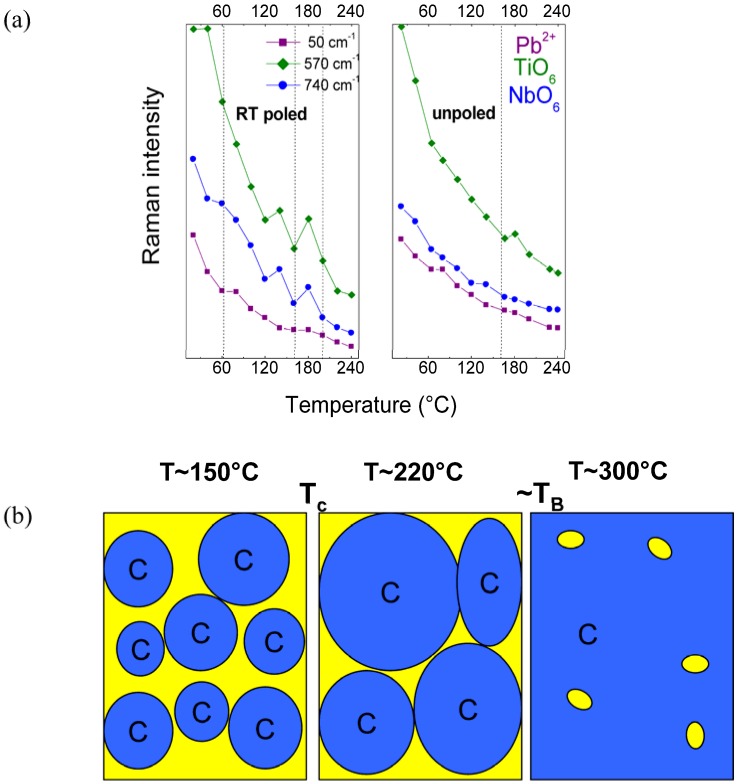
(**a**) Temperature dependences of Raman mode intensities for RT poled and unpoled textured ceramic [62]. The dashed lines represent the phase transition temperature determined from the dielectric and thermal expansion measurements; (**b**) Sketch of continuous evolution of symmetry towards the “true cubic one”(C), *i.e*., cubic without polar nanoregions. T_C_ and T_B_ denote the temperatures of ferro/paraelectric Curie phase transition and the Burns (vanishing of PNR on heating, nucleation on cooling), respectively.

Figure 7a shows the temperature evolution of Raman mode intensities. As it can be clearly seen, the absolute intensity of all analyzed modes decreases significantly with the temperature increase, but there are no drastic changes at the phase transition temperatures. In general, the integrated intensity is proportional to the temperature [27]. Therefore, the shut-down of intensity with increasing temperature can be explained by the changes of the short/long range order correlations as a function of the nanoregion organization. As graphically sketched in Figure 7b, when the temperature increases the local symmetry evolves continuously towards the pure cubic one. Moreover, this fits well with the phase transition determined in the thermal expansion measurements, close to 200 °C [61], and thus shows that the true cubic symmetry, *i.e*., cubic symmetry without polar nanoregions can be achieved only at rather high temperature, *i.e*., much above the maximum of dielectric permittivity. This shows clearly that the Raman intensity as a function of the polarizability and symmetry probes very well the bonding covalence and structure, and thus is a very powerful tool to detect any structural modification [26,27,28,97]. The sketch presented in Figure 7b suggests that the drastic modifications of the ferroelectric properties (near T_C_) appear when coulombic interactions between non-cubic domains are sufficiently lowered and the percolation of cubic domains starts. We can expect that the percolation of cubic domains may be completed in the vicinity of the Burns temperature.

Our results show unambiguously how the temperature dependences of Raman mode parameters can be useful to follow the behavior of different sublattices. Finally, it should be underlined that the values of FWHMs, intensities as well as the wavenumbers show the maximal or minimal values near the phase transitions temperatures, especially those determined by thermal expansion measurements. This shows huge sensibility of both techniques to detect modifications of the chemical bonds.

#### 2.2.2. Temperature evolution of Rayleigh wings

Since the Rayleigh intensity is direct proportional to the dielectric and compositional heterogeneity, at each temperature value the Rayleigh wings, *i.e*., the Raman spectra in both sides of the Rayleigh line, were also recorded.

**Figure 8:**
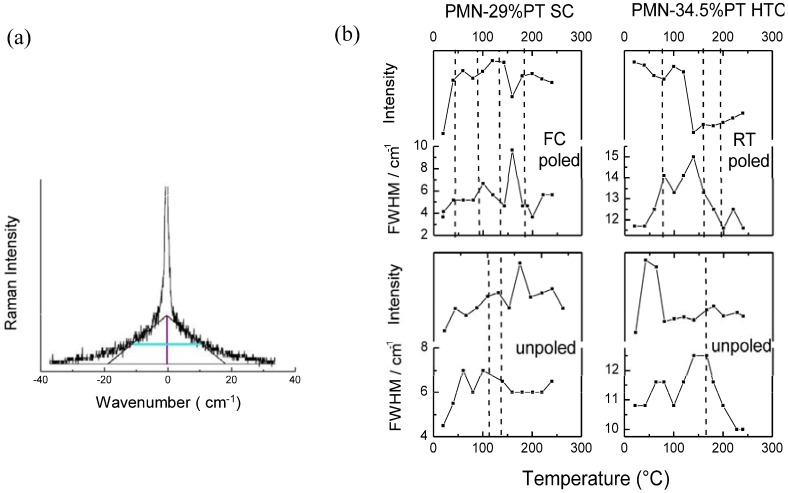
(**a**) Sketch of the so–called triangle method, which allow determination of the intensities and widths of the Rayleigh wings. (**b**) Temperature dependences of the Rayleigh wing intensities and widths for poled and unpoled PMN-29%PT single crystal and PMN-34.5%PT textured ceramic. The dashed lines represent the phase transitions temperature determined from the dielectric and thermal expansion measurements.

It is noteworthy that in the case of relaxors in the vicinity of the Rayleigh line, the central peak can be observed because of the lattice relaxations [62,94,96]. Additionally, in the low wavenumber range another interesting phenomenon is expected, namely the presence of the soft modes. Due to the huge intensity of the Rayleigh peak, very high resolution—higher than that used—is needed for the detection of the low wavenumber components. Inelastic neutron scattering studies [98,99] point to the existence of the ferroelectric soft mode in PMN, which becomes overdamped near the temperature in which the polar nanoregions appear, *i.e*., Burns temperature [52]. Recently, the Raman study [100] has revealed for the first time the presence of soft mode in PMN single crystal. The authors used the angular dependences of Raman spectra in order to eliminate the strong Pb^2+^ mode located near 45 cm^−1^ masking the soft mode signature. This soft mode becomes overdamped in a wide temperature range above T_C_, then recovers the underdamped oscilation and hardens in the high temperature region. The presence of soft mode has not been confirmed by our Raman studies probably due to the nanodomains heterogeneity, which broadens significantly the Rayleigh line. Consequently, the soft mode is overlapped and impossible to detect directly. However, the analysis of the Rayleigh wings allowed us to document the temperature dependences of the intensities and widths for both single crystal and textured ceramic (Figure 8b). Note that the so-called triangle method, in which the Rayleigh peak intensities are normalized and the low wavenumber scattering (Rayleigh wings) is approximated by a triangle (Figure 8a), was used in order to determine these dependences. As it can be seen in Figure 8b, in the vicinity of structural phase transitions determined by dielectric and thermal expansion measurements, the significant step variation of intensity are observed, especially for poled samples. Such important changes of intensity can be associated with the rearrangement of the ferroelectric domain state, which directly influences the optical properties of the material. This shows that the Rayleigh wing intensity that probes the compositional and dielectric heterogeneity appears as sensitive to the nanostructure rearrangement as the temperature dependences of the of Raman wavenumbers.

## 3. Experimental Section

The PMN-29%PT single crystal (SC) from APC (U.S.) was poled by the field cooling method, *i.e*., the crystal was cooled in the presence of an electric field of strength 200V/mm from 170 °C down to −20 °C. The highly textured ceramic (HTC) PMN-34.5%PT, prepared at R&T Thales, was poled at room temperature by applying an electric field of the strength of 800V/mm. The unpoled state for both materials was obtained by heating the samples until a temperature 200 °C higher than the temperature of the maximum of dielectric permittivity. Dielectric measurements were recorded for unpoled and freshly poled samples during heating at a frequency of 1 KHz. Thermal expansion measurements were recorded both in the heating and cooling cycles from −200 °C to 400 °C using a Setsys (Setaram, France) dilatometer equipped with the amorphous silica rod. Raman spectra and Rayleigh wings were recorded from 20 °C to 240 °C in the macro configuration using an XY spectrometer (Dilor, France). The 514.5 nm Ar^+^ laser excitation line perpendicular to the [001] crystallographic direction (both for single crystal and highly textured ceramic) was used. More details can be found in [60,61,62,97].

## 4. Conclusion

Using the example of poled (field cooling or room temperature) and unpoled PMN-PT single crystal and highly textured ceramic from the MPB range we have shown the efficiency of thermal expansion and Raman scattering techniques, methods sensitive to the chemical bond anharmonicity, to detect and to analyze the subtitle structural modifications that is necessary in understanding the complex physical/chemical behavior of this system. The competition between the different sublattices with competing degree of freedom: the Pb-Pb one dominated by the Coulombian interactions as well as NbO_6_ and TiO_6_ ones built of covalent bonded entities, determine the short range arrangement and the outstanding ferro- and piezoelectric properties. It is noteworthy that our studies clearly show the application potential of the textured ceramics and show that they are a low cost alternative to the single crystal.
